# The flavonoid quercetin decreases ACE2 and TMPRSS2 expression but not SARS‐CoV‐2 infection in cultured human lung cells

**DOI:** 10.1002/biof.2084

**Published:** 2024-06-17

**Authors:** Michael James Houghton, Eglantine Balland, Matthew James Gartner, Belinda Jane Thomas, Kanta Subbarao, Gary Williamson

**Affiliations:** ^1^ Department of Nutrition, Dietetics and Food Monash University, BASE Facility Notting Hill VIC Australia; ^2^ Victorian Heart Institute Monash University, Victorian Heart Hospital Clayton VIC Australia; ^3^ Monash Biomedicine Discovery Institute, Department of Anatomy and Developmental Biology Monash University Clayton VIC Australia; ^4^ Department of Microbiology and Immunology University of Melbourne at The Peter Doherty Institute for Infection and Immunity Melbourne VIC Australia; ^5^ Centre for Innate Immunity and Infectious Diseases Hudson Institute of Medical Research Clayton VIC Australia; ^6^ Monash Lung and Sleep, Monash Health, Monash Medical Centre Clayton VIC Australia; ^7^ WHO Collaborating Centre for Reference and Research on Influenza The Peter Doherty Institute for Infection and Immunity Melbourne VIC Australia

**Keywords:** ADAM17, air–liquid interface, apelin‐13, Calu‐3, COVID‐19, estradiol, polyphenol, primary bronchial epithelial cells

## Abstract

Severe acute respiratory syndrome coronavirus 2 (SARS‐CoV‐2) binds to angiotensin‐converting enzyme 2 (ACE2) on host cells, via its spike protein, and transmembrane protease, serine 2 (TMPRSS2) cleaves the spike‐ACE2 complex to facilitate virus entry. As rate‐limiting steps for virus entry, modulation of ACE2 and/or TMPRSS2 may decrease SARS‐CoV‐2 infectivity and COVID‐19 severity. In silico modeling suggested the natural bioactive flavonoid quercetin can bind to ACE2 and a recent randomized clinical trial demonstrated that oral supplementation with quercetin increased COVID‐19 recovery. A range of cultured human cells were assessed for co‐expression of ACE2 and TMPRSS2. Immortalized Calu‐3 lung cells, cultured and matured at an air–liquid interface (Calu‐3‐ALIs), were established as the most appropriate. Primary bronchial epithelial cells (PBECs) were obtained from healthy adult males (*N* = 6) and cultured under submerged conditions to corroborate the outcomes. Upon maturation or reaching 80% confluence, respectively, the Calu‐3‐ALIs and PBECs were treated with quercetin, and mRNA and protein expression were assessed by droplet digital PCR and ELISA, respectively. SARS‐CoV‐2 infectivity, and the effects of pre‐ and co‐treatment with quercetin, was assessed by median tissue culture infectious dose assay. Quercetin dose‐dependently decreased ACE2 and TMPRSS2 mRNA and protein in both Calu‐3‐ALIs and PBECs after 4 h, while TMPRSS2 remained suppressed in response to prolonged treatment with lower doses (twice daily for 3 days). Quercetin also acutely decreased ADAM17 mRNA, but not ACE, in Calu‐3‐ALIs, and this warrants further investigation. Calu‐3‐ALIs, but not PBECs, were successfully infected with SARS‐CoV‐2; however, quercetin had no antiviral effect, neither directly nor indirectly through downregulation of ACE2 and TMPRSS2. Calu‐3‐ALIs were reaffirmed to be an optimal cell model for research into the regulation of ACE2 and TMPRSS2, without the need for prior genetic modification, and will prove valuable in future coronavirus and respiratory infectious disease work. However, our data demonstrate that a significant decrease in the expression of ACE2 and TMPRSS2 by a promising prophylactic candidate may not translate to infection prevention.

## Graphical abstract

Quercetin decreases ACE2, TMPRSS2, and ADAM17 in human Calu‐3 lung cells cultured at an air–liquid interface (Calu‐3‐ALI) but has no effect on severe acute respiratory syndrome coronavirus 2 infection. Created with BioRender.com.

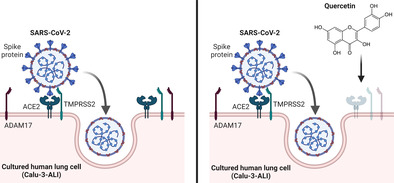

**Abbreviations**
ACE2: angiotensin‐converting enzyme 2
ADAM17: a disintegrin and metalloprotease 17
Estradiol: 17β‐estradiol
FBS: heat‐inactivated fetal bovine serum
Pen/Strep: 100 U/mL penicillin and 100 mg/mL streptomycin
TMPRSS2: transmembrane protease, serine 2

## 1 INTRODUCTION

The COVID‐19 pandemic, caused by severe acute respiratory syndrome coronavirus 2 (SARS‐CoV‐2), instigated an unparalleled global public health crisis. While the development and distribution of vaccines has proven highly efficacious in reducing COVID‐19 severity and SARS‐CoV‐2 transmission, the emergence of new viral variants and the burden of chronic effects after COVID‐19 infection, known as post‐acute sequelae of COVID‐19 or long COVID, underscore the need for ongoing research into effective prophylactic and therapeutic options against this disease.

The SARS‐CoV viruses express a spike protein on their outer surface. The initial mechanism of entry is through interaction of this virus spike protein with membrane‐bound angiotensin‐converting enzyme 2 (ACE2) on the surface of host cells, via interaction between specific amino acid residues. ACE2 is widely expressed in many tissues, including on the lung epithelial cell surface,^1^ and can be released from the cell surface by a disintegrin and metalloprotease 17 (ADAM17) or transmembrane protease, serine 2 (TMPRSS2), two proteases found in proximity to ACE2. Crucially, cleavage of ACE2 by TMPRSS2 after virus spike protein attachment allows SARS‐CoV‐2 to enter cells,^2^ whereas ACE2 cleavage by ADAM17 does not facilitate entry,^3^, ^4^ although ADAM17 may mediate COVID‐19 severity.^5^

ACE2 mutations in the population do not seem to affect binding of the spike protein,^6^ but ACE2 expression is affected by SARS‐CoV‐2 infection and host immune response.^5^ Paradoxically, a high expression of ACE2 could lead to a higher chance of SARS‐CoV‐2 infection but is associated with increased protection against respiratory syndrome and lung injuries.^3^, ^7^ If the target cell expresses insufficient TMPRSS2, or if a virus‐ACE2 complex does not encounter TMPRSS2, the virus‐ACE2 complex is internalized via clathrin‐mediated endocytosis, where spike protein cleavage is performed by cathepsins.^8^, ^9^ However, SARS‐CoV‐2 entry is faster in cells that express TMPRSS2,^8^ and cells that express both ACE2 and TMPRSS2 are more prone to infection.^2^ Further, we demonstrated previously that ACE2 is essential for SARS‐CoV‐2 infection in cultured human stem cell‐derived lung alveolar type II (AT2) epithelial cells^10^ and that ancestral, delta, and omicron (BA.1) SARS‐CoV‐2 strains are dependent on serine proteases for entry throughout the human respiratory tract,^11^ so modulation of one or both of these proteins remains a plausible prophylactic strategy. There remains an unanswered question as to whether a lower expression of ACE2 and/or TMPRSS2 on the surface of nasal and lung cells reduces susceptibility to infection in vivo. It was demonstrated in vitro that ACE2 expression in various cell lines was correlated with susceptibility to SARS‐CoV infection and that soluble ACE2 could act as a chelating agent to inhibit the binding of SARS‐CoV to membrane‐bound ACE2.^12^ If either ACE2 or TMPRSS2 are rate‐limiting steps for virus entry, then there is a convincing case to assess expression of these two proteases as an estimate of infection risk and also a need to search for agents which inhibit the enzyme activities, downregulate expression, or even increase their turnover and/or modify their location.

A variety of drugs and small molecules have been shown to regulate both ACE2 and TMPRSS2 and influence COVID‐19 progression.^13^, ^14^ Inhibitors of ACE2 activity cannot be assumed to inhibit binding of the virus spike protein due to different ligand binding sites and mechanisms. Nevertheless, several (poly)phenols, including quercetin glycosides, can bind to ACE2 according to in silico analyses.^15^ Notably, an in silico study published early in the pandemic identified the flavonoid quercetin from a database of 8000 small molecule candidates as one of the top five most potent compounds for binding to the ACE2‐viral spike protein interface, therefore potentially disrupting viral entry into host cells,^16^ and another study suggested that quercetin could interfere with SARS‐CoV‐2 replication.^17^ A recent review summarized data supporting the notion that quercetin could regulate ACE2 expression.^18^ However, we previously discussed that oral administration of quercetin is unlikely to be effective owing to biotransformation during digestion, absorption, and metabolism, but suggested that it could be administered directly by nasal or throat spray.^19^ Potent inhibitors of TMPRSS2 reduced influenza virus replication in Calu‐3 cells,^20^ while in silico modeling suggests that some flavonoids are possible TMPRSS2 inhibitors.^21^, ^22^ Other molecular docking studies found that quercetin could bind to the SARS‐CoV‐2 spike receptor‐binding domain, with a predicted interaction stronger than remdesivir,^23^ and to the catalytic site of TMPRSS2.^24^

Quercetin has attracted attention for its potential as an antiviral agent against SARS‐CoV‐2. Quercetin, and several other phenolics, attenuated SARS‐CoV‐2 recombinant spike protein‐induced upregulation of ACE2 and TMPRSS2 protein expression in bovine aortic endothelial cells.^25^ The authors hypothesized that this indicated that quercetin might inhibit viral entry into endothelial cells through downregulation of these crucial host entry proteins. The same authors reported that treatment with 17β‐estradiol also inhibited the spike protein‐induced increase in ACE2.^26^ Numerous in silico studies have been performed demonstrating the potential for quercetin to disrupt SARS‐CoV‐2 entry into cells,^27^ but very few in vitro or in vivo studies have demonstrated this. In vitro studies investigating potential prophylactic compounds against SARS‐CoV demonstrated that quercetin inhibited viral entry into simian Vero E6 cells and had the potential to inhibit viral replication.^28^, ^29^ A recent study in humans demonstrated a faster recovery from mild COVID‐19 with oral quercetin supplementation, via increased clearance of SARS‐CoV‐2 and modulation of the hyperinflammatory response.^30^ Early treatment with viral entry inhibitors can be an effective way to reduce COVID‐19.^13^ We hypothesized that quercetin could inhibit entry of the virus into cells, either directly or via regulation of ACE2 and TMPRSS2 expression, thereby impeding the infection, increasing viral clearance and limiting COVID‐19 progression.

In the present study, we aimed to ascertain whether ACE2 and TMPRSS2 were rate‐limiting for SARS‐CoV‐2 viral entry in cultured cells and assess the potential for quercetin to modulate expression of these entry proteins. We have shown previously that SARS‐CoV‐2 entry is dependent on protease entry in physiologically relevant human airway epithelial cell models^11^ and that airway cells cultured at an air–liquid interface (ALI) in particular are useful and practical tools for investigation of SARS‐CoV‐2 infection and evaluating the clinical potential of therapeutics for COVID‐19.^31^ We hypothesized that quercetin could regulate expression of ACE2 and TMPRSS2 at the transcriptomic and/or proteomic levels. We first screened the expression of ACE2 and TMPRSS2 in various human cell lines relevant to SARS‐CoV‐2 infection and COVID‐19, including several airway cell lines and endothelial cells, and explored whether ACE2 and/or TMPRSS2 could be induced by culturing the cells for longer and/or on transmembrane inserts, either submerged or at an ALI. Previously, expression of ACE2 and/or TMPRSS2 was increased during differentiation in polarized cells cultured post‐confluence for longer and/or at an ALI.^32^, ^33^ Using droplet digital PCR (ddPCR), which is sensitive enough to detect lowly expressed genes and subtle changes in gene expression, we assessed gene regulation by exposure to known positive control agents, estradiol^26^, ^34^ and apelin,^35^, ^36^ to determine whether expression could be regulated even in cells with a low basal expression. This could have implications on COVID‐19 progression and treatment, where other cells and tissues are affected beyond the initial SARS‐CoV‐2 respiratory infection. A physiologically relevant Calu‐3‐ALI cell model was selected to assess regulation by quercetin, and this was corroborated in human primary lung cell cultures. Finally, the effect of quercetin on SARS‐CoV‐2 infectivity was assessed using our infectivity assay published previously.^10^, ^11^

## 2 MATERIALS AND METHODS

### 2.1 Materials

Immortalized lung and nasal epithelial cell lines were obtained from the American Type Culture Collection (ATCC) via In Vitro Technologies (Noble Park North, VIC Australia), unless otherwise stated. Quercetin dihydrate (purity ≥99% by HPLC) was from Extrasynthese (Genay, France) and solubilized in DMSO. All cell culture medium components, culture vessels (manufactured by Corning), and other chemicals and reagents were purchased from Merck Life Science (Bayswater, VIC, Australia) unless otherwise stated. Ultrapure water (18.2 MΩ/cm) supplied by a MilliQ system (Merck Life Science) was used throughout unless otherwise stated. β‐estradiol was solubilized initially in ethanol and diluted in H_2_O, with a final ethanol concentration in culture medium <0.003% (v/v). Apelin‐13 trifluoroacetate salt was solubilized in H_2_O. FAM‐ or VIC‐labeled TaqMan primers for ACE2 (Hs01085333_m1), TMPRSS2 (Hs01122322_m1), ACE (Hs00174179_m1), ADAM17 (Hs01041915_m1), TBP (Hs00427620_m1), GAPDH (Hs99999905_m1), and ACTB (Hs99999903_m1) were purchased from Thermo Fisher Scientific (Scoresby, VIC, Australia). The QX200 ddPCR system, ddPCR Supermix for Probes (no dUTP), and all other materials used for ddPCR were from Bio‐Rad Laboratories (South Granville, NSW, Australia).

### 2.2 Ethics approval

Primary human bronchial epithelial cells were obtained from normal subjects whom were non‐smokers or had not smoked for >15 years, and none had a diagnosis of asthma or chronic obstructive pulmonary disease (normal FEV_1_ measurements). Studies were approved by the Monash Health and Monash Medical Centre Research Ethics Committee (HREC #02052A), consent was obtained from all subjects, and studies were conducted in accordance with the approved guidelines.

### 2.3 Cell culture

All cells were maintained in a humidified atmosphere of 5% CO_2_/95% air at 37°C and passaged in 75 cm^2^ flasks. When passaging and seeding cells for experiments, cells were counted and viability was monitored by Trypan Blue exclusion assay using a TC10 automated cell counter (Bio‐Rad Laboratories, Hercules, CA, USA). Where culture media was supplemented with 10% (v/v) heat‐inactivated fetal bovine serum, this is referred to simply as FBS and supplementation with 100 U/mL penicillin and 100 mg/mL streptomycin is referred to simply as Pen/Strep.

#### 2.3.1 Human umbilical vein endothelial cell

Primary human umbilical vein endothelial cells (HUVEC) were purchased from Lonza (Cat. C2519A, Basel, Switzerland) and cultured in endothelial growth medium 2 supplemented with endothelial growth medium 2 bullet kit (Lonza Australia, Sydney, NSW, Australia). Cells were used between passages 3–6.

#### 2.3.2 NCI‐H23

Immortalized human lung adenocarcinoma NCI‐H23 cells (ATCC CRL‐5800; RRID:CVCL_1547) were cultured in RPMI‐1640 medium (Thermo Fisher Scientific) supplemented with FBS and Pen/Strep.

#### 2.3.3 A549

Immortalized human lung carcinoma A549 cells (ATCC CCL‐185; RRID:CVCL_0023) were cultured in Ham's F‐12K (Kaighn's) medium (Thermo Fisher Scientific) supplemented with FBS and Pen/Strep.

#### 2.3.4 RPMI 2650

Immortalized human nasal carcinoma RPMI 2650 cells (ATCC CCL‐30; RRID:CVCL_1664) were cultured in Eagle's minimum essential medium (ATCC, In Vitro Technologies) supplemented with FBS and Pen/Strep.

#### 2.3.5 Calu‐3

Immortalized human lung adenocarcinoma Calu‐3 cells (ATCC HTB‐55; RRID:CVCL_0609) were cultured in Eagle's minimum essential medium (Merck Life Science) supplemented with FBS, Pen/Strep, 2% (v/v) non‐essential amino acids and 1% (v/v) sodium pyruvate.

#### 2.3.6 Vero and VeroE6/TMPRSS2

African green monkey kidney epithelial Vero cells (ATCC CCL‐81, RRID:CVCL_0059) and Vero E6 cells expressing TMPRSS2 (Japanese Collection of Research Bioresources [JCRB] Cell Bank 1819, Osaka, Japan; CVCL_YQ49) were cultured in Minimum Essential Media (MEM, Media Preparation Unit, Peter Doherty Institute) supplemented with 5% (v/v) FBS, Pen/Strep, 2 mM GlutaMAX (Thermo Fisher Scientific) and 15 mM HEPES (Thermo Fisher Scientific).

#### 2.3.7 Vero/hSLAM

Vero cells expressing human signaling lymphocytic activation molecule (Vero/hSLAM, Cat. 04091501, European Collection of Authenticated Cell Cultures [ECACC], Porton Down, UK; RRID:CVCL_L037) were cultured in MEM supplemented with 7% (v/v) FBS, Pen/Strep, 2 mM GlutaMAX, 15 mM HEPES, and 0.25 mg/mL Geneticin (Thermo Fisher Scientific).

#### 2.3.8 Human primary bronchial epithelial cell culture

Human primary bronchial epithelial cells (PBECs) were obtained from bronchial brushings and cultured under submerged conditions on collagen‐coated flasks (MP Biochemicals, Santa Ana, CA, USA) in bronchial epithelial growth media (BEGM; Lonza Australia).

### 2.4 Virus

hCoV‐19/Australia/VIC01/2020 (GenBank: MT007544.1) was a gift obtained from the Victorian Infectious Diseases Reference Laboratory. SARS‐CoV‐2 stocks were propagated in Vero/hSLAM cells in serum‐free MEM in the presence of 1 μg/mL TPCK‐trypsin (Worthington Biochemical via ScimaR, Brighton, VIC, Australia). Virus stocks were harvested at 70%–90% cytopathic effect in cultures, centrifuged at 500× *g* for 5 min to remove cellular debris, aliquoted into cryovials, and stored at −80°C. Stocks were titred as described below.

### 2.5 Cell experiments

Cells were seeded for gene and protein expression experiments on either solid flat‐bottomed six‐well plates or on 24 mm Transwell polycarbonate membrane inserts (0.4 μm pore size) in six‐well plates, while, for SARS‐CoV‐2 infectivity assays, PBECs were cultured on solid flat‐bottomed 24‐well plates and Calu‐3 cells were cultured on 6.5 mm Transwell polycarbonate membrane inserts (0.4 μm pore size) in 24‐well plates. Upon reaching confluence, cells on transmembrane inserts were maintained on the submerged transmembrane support (STM), with media in both apical and basolateral compartments, or the media in the apical compartment was removed and cells were maintained at a physiologically relevant ALI, as done previously.^37^, ^38^, ^39^, ^40^ Cells were maintained in one of these three formats for up to 20 days post‐confluence and media was changed every 2–3 days, with the final media change always 24 h prior to each experiment. It was confirmed by ddPCR that co‐expression of ACE2 and TMPRSS2 was highest in Calu‐3 cells differentiated at an ALI (Calu‐3‐ALIs) (Table 1) so this cell model was used for further experiments. Trans‐epithelial electrical resistance (TEER) was measured using a Millicell ERS voltohmmeter (Millipore, Merck Life Science, Watford, UK) according to the manufacturer's instructions to confirm that TEER values of Calu‐3‐ALIs were similar to those reported in the literature previously.^38^, ^40^ TEER values and cell viability (Trypan Blue exclusion assay) of Calu‐3‐ALIs were also checked following quercetin treatment (Table S1).

**TABLE 1:** Angiotensin‐converting enzyme 2 (ACE2) and transmembrane protease, serine 2 (TMPRSS2) expression in human cell lines and effects of culture time and format, and response to estradiol or apelin‐13.

Cell line	Culture conditions		Treatment^c^	Expression^d^	
	Time (days)^a^	Format^b^		ACE2	TMPRSS2
HUVEC	0	Solid	Untreated	0.57 ± 0.28	0.66 ± 1.40
	3		Control	0.50 ± 0.16	0.10 ± 0.07
			Estradiol	0.71 ± 0.15	0.23 ± 0.32
	7		Control	0.86 ± 0.45	0.29 ± 0.32
			Estradiol	0.64 ± 0.18	0.15 ± 0.10
NCI‐H23	0	Solid	Control	1.32 ± 0.20	47.0 ± 7.3
			Estradiol	0.88 ± 0.18	34.0 ± 6.0
			Apelin‐13	0.93 ± 0.11	37.4 ± 4.2
	5		Control	1.09 ± 0.15	39.2 ± 12.8
			Estradiol	1.04 ± 0.28	37.9 ± 9.0
			Apelin‐13	1.29 ± 0.35	39.6 ± 4.1
	10		Control	2.32 ± 0.43*	42.6 ± 10.3
			Estradiol	2.02 ± 0.91	36.8 ± 13.2
			Apelin‐13	2.72 ± 0.53	46.3 ± 10.5
	15		Control	13.9 ± 2.7**	60.5 ± 9.6*
			Estradiol	15.8 ± 0.8***	85.4 ± 8.9***^##^
			Apelin‐13	15.0 ± 2.7***	75.3 ± 15.6***
A549	0	Solid	Control	2.82 ± 1.54	2.56 ± 1.54
			Estradiol	1.93 ± 0.76	2.06 ± 1.19
			Apelin‐13	2.56 ± 1.47	3.53 ± 1.95
	5		Control	1.85 ± 1.52	6.47 ± 3.56
			Estradiol	1.66 ± 0.87	6.93 ± 3.96
			Apelin‐13	2.60 ± 2.37	7.81 ± 4.95
	10		Control	1.46 ± 1.14	14.3 ± 16.7
			Estradiol	1.47 ± 0.87	9.31 ± 7.50
			Apelin‐13	2.26 ± 2.13	14.7 ± 10.3*
	15		Control	3.26 ± 4.41	31.4 ± 29.2***
			Estradiol	5.25 ± 6.62	32.4 ± 31.9***
			Apelin‐13	2.55 ± 5.39	18.5 ± 9.5*
RPMI 2650	0	Solid	Control	0.42 ± 0.20	23.3 ± 7.6
			Estradiol	1.07 ± 0.28	43.3 ± 5.0
			Apelin‐13	0.90 ± 0.27	30.8 ± 0.8
	10	Solid	Control	0.52 ± 0.12	10.8 ± 0.6
			Estradiol	1.10 ± 0.05	21.6 ± 4.6
			Apelin‐13	0.91 ± 0.15	14.3 ± 1.9
		STM	Control	0.43 ± 0.23	7.05 ± 1.87
			Estradiol	0.96 ± 0.14	6.21 ± 1.79**
			Apelin‐13	1.12 ± 0.40	12.5 ± 1.4
		ALI	Control	0.32 ± 0.16	7.22 ± 4.45
			Estradiol	1.02 ± 0.24	8.45 ± 1.18
			Apelin‐13	0.69 ± 0.10	9.70 ± 0.38
	20	Solid	Control	0.70 ± 0.49	5.19 ± 4.02*
			Estradiol	1.75 ± 0.15	13.3 ± 3.0
			Apelin‐13	1.19 ± 0.45	6.92 ± 2.58
		STM	Control	0.26 ± 0.13	2.10 ± 0.98***
			Estradiol	0.59 ± 0.29*	5.98 ± 1.63**
			Apelin‐13	1.11 ± 0.23	6.87 ± 1.27
		ALI	Control	0.49 ± 0.06	3.17 ± 0.38**
			Estradiol	0.48 ± 0.34*	8.21 ± 2.71
			Apelin‐13	1.01 ± 0.20	10.5 ± 3.1
Calu‐3	0	Solid	Control	671 ± 122	165 ± 58
			Estradiol	611 ± 54	153 ± 8
			Apelin‐13	757 ± 98	195 ± 25
	10	Solid	Control	747 ± 107	134 ± 28
			Estradiol	900 ± 147	167 ± 21
			Apelin‐13	660 ± 78	125 ± 16*
		STM	Control	2188 ± 438**	107 ± 27
			Estradiol	2260 ± 243***^§§^	143 ± 22
			Apelin‐13	2453 ± 540***^§§^	209 ± 34^§###^
		ALI	Control	2937 ± 94***	228 ± 24***
			Estradiol	3135 ± 729***^§§^	218 ± 36*
			Apelin‐13	2945 ± 134***^§§§^	215 ± 7^§§§^
	20	Solid	Control	839 ± 120^††^	143 ± 28^§§^
			Estradiol	959 ± 208*^††^	162 ± 42
			Apelin‐13	1089 ± 394^†^	164 ± 50
		STM	Control	2576 ± 310**	99.0 ± 17**^§§§^
			Estradiol	3766 ± 588***^§§§^	194 ± 22^###^
			Apelin‐13	3541 ± 303***^§§§#^	191 ± 13^§###^
		ALI	Control	3995 ± 956**	139 ± 25^§§^
			Estradiol	4311 ± 453***^§§§†^	229 ± 41**^##^
			Apelin‐13	4544 ± 291***^§§§†††^	223 ± 24^§§§##^

*Note*: Statistical analyses for the above table. Human umbilical vein endothelial cells (HUVEC; *n*/*N* = 6/3): no significant differences in ACE2 or TMPRSS2 detected by Friedman tests. NCI‐H23 (*n*/*N* = 6/3): significant main effects of culture time on ACE2 and TMPRSS2 (both *p* < 0.001), and a significant culture time × treatment interaction for TMPRSS2 (*p* < 0.001), detected by repeated‐measures two‐way ANOVA (with the Geisser–Greenhouse correction applied to ACE2 data) with post‐hoc Tukey's multiple comparisons: ACE2 in control cells *p < 0.05 vs. Days 0 and 5; **p < 0.01 Days 0, 5, and 10. ACE2 in estradiol‐treated cells ***p < 0.001 vs. Days 0, 5, and 10. ACE2 in apelin‐13‐treated cells ***p < 0.001 vs. Day 0. TMPRSS2 in control cells *p < 0.05 vs. Days 5 and 10. TMPRSS2 in estradiol‐treated cells ***p < 0.001 vs. Days 0, 5, and 10; ^##^ *p* < 0.01 vs. control. TMPRSS2 in apelin‐13‐treated cells ***p < 0.001 vs. Day 0.
A549 (*n*/*N* = 12/3): no significant differences in ACE2 but significant differences in TMPRSS2 (*p* < 0.001) detected by Friedman tests with post‐hoc Dunn's multiple comparisons: TMPRSS2 in control cells ***p < 0.001 vs. Day 0. TMPRSS2 in estradiol‐treated cells ***p < 0.001 vs. Day 0. TMPRSS2 in apelin‐13‐treated cells *p < 0.05 vs. Day 0.
RPMI 2650 (*n*/*N* = 6/3): significant differences in ACE2 and TMPRSS2 (both *p* < 0.001) detected by Friedman tests with post‐hoc Dunn's multiple comparisons: ACE2 in estradiol‐treated cells *p < 0.05 vs. Day 20 solid. TMPRSS2 in control cells *p < 0.05 vs. Day 0; **p < 0.01, ***p < 0.001 vs. Day 0 and Day 10 solid. TMPRSS2 in estradiol‐treated cells **p < 0.01 vs. Day 0.
Calu‐3 (*n*/*N* = 6/3): significant differences in log‐transformed ACE2 and TMPRSS2 data (both *p* < 0.001) detected by repeated‐measures one‐way ANOVA (with the Geisser–Greenhouse correction applied to ACE2 data) with post‐hoc Tukey's multiple comparisons: ACE2 in control cells **p < 0.01, ***p < 0.001 vs. Day 0 and Day 10 solid; ^††^ *p* < 0.01 vs. Days 10 and 20 STM and ALI. ACE2 in estradiol‐treated cells *p < 0.05, ***p < 0.001 vs. Day 0; ^§§^ *p* < 0.01, ^§§§^ *p* < 0.001 vs. Day 10 solid; ^†^ *p* < 0.05 vs. Day 10 STM; ^††^ *p* < 0.01 vs. Days 10 and 20 STM and ALI. ACE2 in apelin‐13‐treated cells ***p < 0.001 vs. Day 0; ^§§^ *p* < 0.01, ^§§§^ *p* < 0.001 vs. Day 10 solid; ^†^ *p* < 0.05 vs. Day 10 ALI and Day 20 STM and ALI; ^†††^ *p* < 0.001 vs. Day 10 ALI; ^#^ *p* < 0.05 vs. control. TMPRSS2 in control cells **p < 0.01 vs. Day 0; ***p < 0.001 vs. Day 10 solid and STM; ^§§^ *p* < 0.01, ^§§§^ *p* < 0.001 vs. Day 10 ALI. TMPRSS2 in estradiol‐treated cells *p < 0.05, **p < 0.01 vs. Day 10 STM; ^##^ *p* < 0.01, ^###^ *p* < 0.001 vs. control.
TMPRSS2 in apelin‐13‐treated cells *p < 0.05 vs. Day 0; ^§^ *p* < 0.05, ^§§§^ *p* < 0.001 vs. Day 10 solid; ^##^ *p* < 0.01, ^###^ *p* < 0.001 vs. control.

^a^ Days in culture since reaching confluence.

^b^ Cells were cultured on a solid well plate, on a submerged transmembrane support (STM) or at an air–liquid interface (ALI).

^c^ Cells were treated for 4 h with 100 nM estradiol, 100 nM apelin‐13, or 0.1% DMSO (v/v) vehicle control.

^d^ Mean ± SD absolute copy number per ng mRNA, quantified by droplet digital PCR. With large numbers of multiple comparisons (210 for RPMI 2650 and Calu‐3), in the cases where some statistically significant differences overlapped, the largest *p*‐value is shown.

For gene and protein expression, cells were starved of serum for 4 h (basolateral compartment only for cells cultured at an ALI) and then treated for up to 8 h with 100 nM estradiol, 100 nM apelin‐13, quercetin (5, 10, or 20 μM), or 0.1% DMSO (v/v) vehicle control. Two time‐points (3 and 6 h) were selected for the PBEC experiments to maximize the chance of detecting subtle changes in gene expression, while 4 h was selected for gene expression experiments on the HUVEC and immortalized airway cells. We have demonstrated that quercetin is effective at regulating gene expression during this timeframe previously.^41^ Following treatments, cells were washed with PBS and collected for RNA or protein analysis accordingly, with samples stored at −80°C until further processing and analysis.

Longer‐term treatments with quercetin on Calu‐3‐ALIs were performed by treating apically with quercetin (5 or 10 μM) or 0.1% (v/v) DMSO vehicle control in serum‐free medium for 1 h twice per day for the final 3 days of culture. Following each 1 h treatment, the quercetin‐containing medium was aspirated from the apical compartment to return the cells to the ALI format. The final treatment was started simultaneously with serum removal from the basolateral compartment so that cells were serum‐starved for the final 4 h to match the acute experiments; cells were maintained for 3 h with serum‐free medium in the basolateral compartment after the final apical quercetin application was removed and then washed with PBS and collected for RNA or protein analysis.

For SARS‐CoV‐2 infectivity assays, an initial experiment was performed to check feasibility and conditions of PBEC and Calu‐3‐ALI infectivity. Calu‐3‐ALIs, but not PBECs, were successfully infected, as we have shown previously,^42^ and therefore used for further experiments. We set out to assess whether the longer‐term pre‐treatment with quercetin (i.e., effects on gene regulation of entry proteins) and/or co‐treatment with quercetin (i.e., direct effects on the virus and/or virus spike protein‐host entry protein interaction) during inoculation could affect SARS‐CoV‐2 infectivity. Longer‐term treatments were performed by treating Calu‐3‐ALIs apically with quercetin (5 or 10 μM) or 0.1% (v/v) DMSO vehicle control in serum‐free medium for 1 h twice per day for the final 3 days of culture and then, 3 h after the final quercetin application was removed, the cells were inoculated with SARS‐CoV‐2. Short‐term treatments were performed by treating Calu‐3‐ALIs apically with quercetin (5 or 10 μM) or 0.1% (v/v) DMSO vehicle control in serum‐free medium for 1 h with the SARS‐CoV‐2 inoculum. The combined treatments were performed by following the long‐term treatment regimen but with quercetin re‐added to the cells with the inoculum, as done for the short‐term treatment. The control treatment had only the vehicle control applied throughout.

### 2.6 RNA isolation and reverse transcription

Following treatment and PBS wash, total RNA was isolated from Calu‐3 cells using the Aurum Total RNA Mini Kit (Bio‐Rad Laboratories, South Granville, NSW, Australia), following the manufacturer's instructions. Cells were collected in the Total RNA Lysis Solution, supplemented with 1% (v/v) β‐mercaptoethanol, and stored at −80°C. RNA was purified on silica membranes using the spin column‐mediated protocol, including DNase I digest. RNA content and purity were determined spectrophotometrically at 260 nm on a NanoDrop (Thermo Fisher Scientific, Waltham, MA, USA) and cDNA was synthesized by reverse transcription from 1 μg RNA, diluted with RNase/DNase‐free water (Thermo Fisher Scientific) using the High Capacity RNA‐to‐cDNA kit (Thermo Fisher Scientific), following the manufacturer's guidelines. Reactions were incubated at 37°C for 1 h in a C1000 Touch thermal cycler (Bio‐Rad Laboratories) and stored at −20°C until ready for analysis by ddPCR.

### 2.7 Gene expression assays by ddPCR

The QX200 ddPCR system was used to quantify changes in gene expression as previously described for the QX100 system.^41^ Primers were duplexed (one FAM‐labeled and one VIC‐labeled per reaction) whereby a target gene and a reference gene were quantified simultaneously in each assay. ACE2, TMPRSS2, ACE, and ADAM17 genes were probed as the target genes of interest, while TBP, GAPDH, and ACTB were used as reference genes, acting as internal controls to account for variation in liquid handling, droplet generation, and/or PCR efficiency and to account for biological variation across passages and in response to cell treatments. Primers and cDNA were mixed and diluted accordingly with RNase/DNase‐free water in a 20 μL assay that contained 10 μL ddPCR Supermix. The mixtures were partitioned into oil droplets using the QX200 Droplet Generator and then transferred to the C1000 Touch thermal cycler. Droplet‐containing mixtures were incubated for 10 min at 95°C, followed by 40 cycles of 30 s at 94°C and 1 min at 57.8°C, and finished with 10 min incubation at 98°C. Products were maintained at 12°C before analysis on the QX200 Droplet Reader (mean accepted partitions = 16,860 ± 1801; *n* = 1068).

The QuantaSoft software (Bio‐Rad Laboratories, Košice, Slovakia) was used to analyze the data and determine concentrations of the target or reference DNA in copies/μL from the fraction of positive reactions using Poisson distribution analysis. Data were collected independently for each target and reference and are presented as copies per ng mRNA or target gene expression relative to reference gene, assuming a 1:1 RT‐PCR efficiency. ddPCR experiments were designed and performed according to the dMIQE guidelines,^43^ with positive (e.g., samples analyzed previously) and negative (e.g., reverse transcriptase‐free, template‐free, amplification‐free, primer‐free) controls run in each experiment accordingly.

### 2.8 Protein quantification by ELISA

Following treatment and PBS wash, duplicate Calu‐3‐ALIs (one for ACE2 and one for TMPRSS2) were snap‐frozen in situ. ELISAs were performed within 7 days of cell experiments. For ACE2 protein expression, the human ACE2 SimpleStep ELISA kit (ab235649, Abcam, Cambridge, UK) was used, following the manufacturer's instructions. Briefly, frozen cell samples were solubilized in chilled cell extraction buffer for 15 min on ice. Lysates were collected and centrifuged at 18,000× *g* at 4°C for 20 min and the supernatants were assayed immediately, with an aliquot reserved for total protein measurement later by BCA assay (Thermo Fisher Scientific). Samples and standards (recombinant human ACE2 protein) were added to the 96‐well ELISA plate and incubated with a mix of human ACE2 capture and detector antibodies for 1 h on a shaker at RT. After incubation, unbound material was removed with washing. TMB (3,3′,5,5′‐tetramethylbenzidine) solution was added and incubated with intermittent shaking at RT in a PheraSTAR FS microplate reader (BMG LabTech, Ortenberg, Germany) so the development of the blue coloration could be monitored at 600 nm. After 5 min, the reaction was stopped by addition of the stop solution and endpoint absorbance at 450 nm recorded. ACE2 protein data were corrected for total protein.

TMPRSS2 protein expression was quantified by the human TMPRSS2 ELISA kit (NBP2‐89171, Novus Biologicals, supplied by In Vitro Technologies). Frozen Calu‐3‐ALI samples were collected in chilled PBS, freeze‐thawed three times, and passed through a needle (24G, Becton Dickinson, Macquarie Park, NSW, Australia) to disrupt the cells. Lysates were centrifuged at 1500× *g* at 4°C for 10 min and the supernatants were assayed immediately, again with an aliquot reserved for BCA total protein assay. Samples and standards (recombinant human TMPRSS2 protein) were added to the 96‐well ELISA plate, which was pre‐coated with primary antibody, and incubated for 90 min at 37°C. Unbound material was discarded (without washing), biotinylated detection antibody added and the plate incubated for a further 60 min at 37°C. Unbound material was removed with washing and wells were incubated with HRP conjugate solution for 30 min at 37°C, before a second round of washing. Substrate solution was added and the plate was incubated at 37°C in the PheraSTAR FS plate reader, while luminescence settings were optimized and luminescence endpoint recorded, all of which was achieved within <5 min. TMPRSS2 protein data were corrected for total protein.

### 2.9 Virus titration assays to assess SARS‐CoV‐2 infectivity

Virus titrations were performed in 96‐well plates with confluent Vero and VeroE6/TMPRSS2 monolayers. Cells were washed with fresh plain MEM, and this was replaced with 180 μL serum‐free media containing 1 μg/mL TPCK‐Trypsin. Each sample was titrated in quadruplicate by adding 20 μL of supernatant to the first well and performing 10‐fold serial dilutions. Cells were incubated at 37°C and assessed microscopically for SARS‐CoV‐2‐induced cytopathic effect on day 4. Virus titers are expressed as mean log_10_ tissue culture infectious dose (TCID) per mL (TCID_50_/mL).

### 2.10 Virus infection assays

Calu‐3‐ALIs were inoculated with 10^3^ or 10^4^ 50% TCID (TCID_50_) of SARS‐CoV‐2/VIC01 for 1 h at 37°C. The inoculum was removed and the apical surface washed twice with PBS, with the second wash collected as the day 0 sample. Every 2 days, for up to 12 days post‐infection, 500 μL PBS was added to the apical chamber and incubated at 37°C for 10 min, then removed and stored at −80°C. Samples were assayed for infectious virus by virus titration, as described above.

### 2.11 Statistical analyses

Data that were dependent on more than one independent variable (e.g., culture time/format and cell treatment) and followed a Gaussian distribution, assessed by Shapiro–Wilk normality tests, were analyzed by two‐way ANOVA with repeated measures. Data were matched across the same biological passage/experiment and the Geisser–Greenhouse correction was applied to data where homogeneity of variance (assessed by the Brown‐Forsythe and Spearman's rank correlation tests) and sphericity (evaluated by Mauchly's test epsilon) could not be assumed. Significant main effects were analyzed using post‐hoc Tukey's multiple comparisons tests. Data that were not normally distributed, even when log‐transformed, or dependent on only one independent variable, were analyzed by one‐way ANOVA with repeated measures, with the Geisser–Greenhouse correction applied to data where homogeneity and sphericity could not be assumed, and with post‐hoc Tukey's multiple comparisons. Where the residuals of the one‐way ANOVA did not follow a normal distribution, data were analyzed instead by nonparametric Friedman tests with post‐hoc Dunn's multiple comparisons. Statistical analyses were performed, and figures constructed, using GraphPad Prism 9 (Boston, MA, USA).

## 3 RESULTS

### 3.1 Expression of ACE2 and TMPRSS2 in cultured cells

A range of cultured human cells relevant to SARS‐CoV‐2 infection (i.e., airway) and/or COVID‐19 pathophysiology (i.e., vasculature) were assessed for expression of host entry proteins ACE2 and TMPRSS2 at the mRNA level, and expression was regulated by estradiol and apelin‐13, as expected (Table 1). Expression of ACE2 was negligible (though just detectable by ddPCR) in human primary endothelial (HUVEC) and immortalized nasal epithelial (RPMI 2650) cells, regardless of culture time or format, and was uninducible by estradiol or apelin‐13. Of the human immortalized lung epithelial cells that were screened, ACE2 expression was very low in A549 cells, and in NCI‐H23 cells unless cultured for 15 days, and again was unaffected by estradiol or apelin‐13. However, ACE2 was highly expressed in Calu‐3 cells, especially when cultured on a transmembrane support, whether submerged (STM) or at an ALI. The highest levels of ACE2 mRNA were detected in Calu‐3 cells cultured for 20 days at an ALI; this was greater than when cultured for 10 days on a STM (in cells treated with estradiol for 4 h) (*p* < 0.05) or for 10 days at an ALI (in cells treated with apelin‐13 for 4 h) (*p* < 0.001).

TMPRSS2 expression was negligible in HUVEC but expressed in all airway cells to varying degrees. NCI‐H23 cells expressed more TMPRSS2 than A549 or RPMI 2650 cells, and, like ACE2, this was highest on day 15 post‐confluence and was increased by estradiol after 4 h (*p* < 0.01). TMPRSS2 was also highest in A549 cells on day 15, while in RPMI 2650 cells, expression decreased over time. Calu‐3 cells exhibited the highest expression of TMPRSS2, though, unlike ACE2 in Calu‐3, expression was not affected by culturing the cells for longer or in different formats to the same degree. In the control cells, TMPRSS2 was highest in cells cultured for 10 days at an ALI (*p* < 0.001 vs. solid and STM on day 10) but this significantly decreased in cells at an ALI 10 days later (*p* < 0.01) and culturing on a STM for 20 days resulted in the lowest expression. Exposure to estradiol for 4 h significantly upregulated TMPRSS2 in cells cultured on a STM (*p* < 0.001) or at an ALI (*p* < 0.01) for 20 days, while apelin‐13 increased expression in cells cultured on a STM for 10 days (*p* < 0.001) or on a STM (*p* < 0.001) or at an ALI (*p* < 0.01) for 20 days. The effect was pronounced in the STM cells since culturing at an ALI already augmented TMPRSS2.

Treating immortalized airway cell lines with estradiol or apelin‐13 regulated expression of ACE2 and TMPRSS2, as expected. Estradiol increased the expression of TMPRSS2 after 4 h in NCI‐H23 cells that had been cultured for 15 days (*p* < 0.01) and in Calu‐3 cells cultured for 20 days on STM (*p* < 0.001) or at an ALI (*p* < 0.01), while enhancing the upregulation of ACE2 in Calu‐3 cells cultured for longer (day 20 solid vs. day 0, *p* < 0.05). Exposure to apelin‐13 for 4 h upregulated ACE2 in Calu‐3 cells cultured on a STM for 20 days (*p* < 0.05) and upregulated TMPRSS2 in Calu‐3 cells cultured on a STM for 10 or 20 days (both *p* < 0.001) or at an ALI for 20 days (*p* < 0.01). It is worth noting that most effects of the positive control cell treatments in each experiment were subtle compared to the effects of culture time and format and lost statistical significance due to the highly adjusted *p* values caused by the large number of comparisons (210 comparisons for RPMI 2650 and Calu‐3). The regulation of ACE2, and notably TMPRSS2, expression by the positive controls further verified Calu‐3 as the most suitable cell model from those assessed for studying the potential effects of quercetin on the expression of the SARS‐CoV‐2 host entry proteins.

### 3.2 Effects of acute quercetin treatment on expression of ACE2 and TMPRSS2 in Calu‐3‐ALIs

**FIGURE 1:**
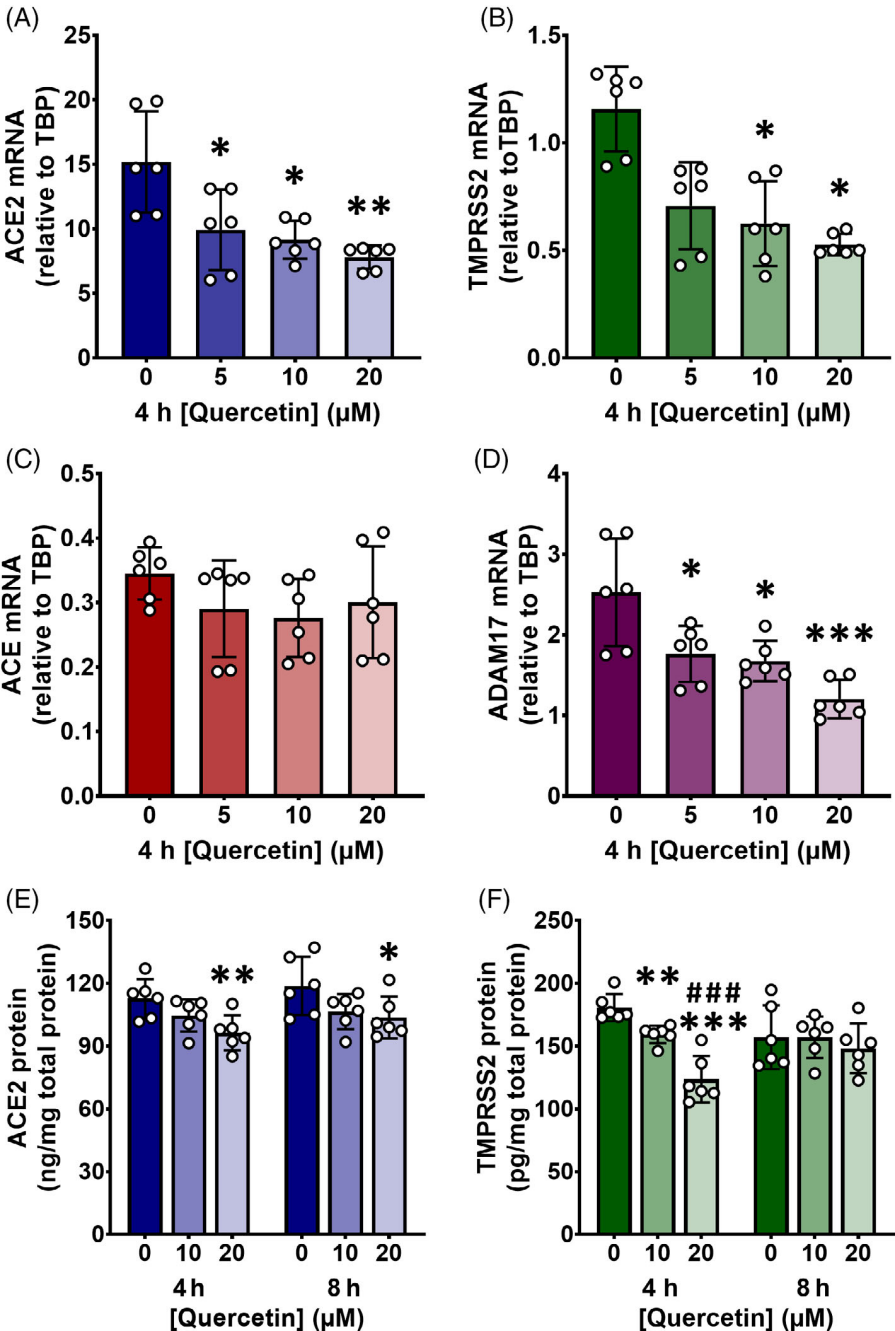
Quercetin acutely decreases angiotensin‐converting enzyme 2 (ACE2) and transmembrane protease, serine 2 (TMPRSS2) in Calu‐3‐air–liquid interface (ALI) cells. Calu‐3 cells were cultured on polycarbonate membrane inserts at an ALI for 10 days after reaching confluence and treated apically with quercetin or 0.1% (v/v) DMSO vehicle control in serum‐free medium for 4 or 8 h. For gene expression (A–D), total RNA was isolated and cDNA was synthesized, partitioned, amplified, and analyzed by ddPCR using gene expression assays. Gene expression was quantified as copies per ng mRNA, assuming a 1:1 RT efficiency, and ACE2 (A), TMPRSS2 (B), ACE (C), and ADAM17 (D) target genes are expressed relative to the TBP reference gene. For protein expression (E, F), ACE2 (E) and TMPRSS2 (F) were quantified by ELISA and expressed relative to total protein in the cell lysate. Data were collected independently for each gene/protein from cells treated on duplicate plates across three biological passages. Data are presented as mean ± SD with individual data points representing each cell experiment (*n*/*N* = 6/3). Significant differences were determined by one‐way (A–C) or two‐way (E, F) ANOVA with post‐hoc Tukey's multiple comparisons or by Friedman test with post‐hoc Dunn's multiple comparisons where residuals did not follow a normal distribution (D). *p < 0.05, **p < 0.01, ***p < 0.001 vs. 0 μM quercetin; ^###^ *p* < 0.001 vs. 10 μM quercetin.

Calu‐3 cells were the most appropriate model for investigating regulation of ACE2 and TMPRSS2, and culturing the cells for 10 or 20 days at an ALI was optimal (Table 1). Quercetin dose‐dependently decreased ACE2 (*p* = 0.0044), TMPRSS2 (*p* = 0.0019), and ADAM17 (*p* = 0.0008), but not ACE (*p* = 0.4526), relative to TBP at the transcriptomic level after 4 h in Calu‐3 cells cultured at an ALI for 10 days (Figure 1A–D). Absolute expression of ACE2, TMPRSS2, and ADAM17 was also dose‐dependently decreased, while TBP was unchanged (Table S2). Gene expression assays for ACE2 and TMPRSS2 were first conducted on Calu‐3 cells cultured at an ALI for 20 days, since ACE2 expression was at its highest in this model (Table 1) and quercetin dose‐dependently decreased expression of both (Table S2), corroborating the results seen in cells cultured for 10 days. However, since there was no difference in the results between the experiments performed on cells cultured for 20 or 10 days, and it was more practical to culture cells for only 10 days, experiments were continued using the latter. Notably, estradiol and apelin‐13 had no effect on ACE2 or TMPRSS2 in day 10 ALI cells and significantly increased TMPRSS2 in day 20 ALI cells (Table 1), so it is likely that the quercetin‐induced changes were caused by another mechanism.

TBP was selected as the reference gene because, of those screened, its expression was closest in range to that of both ACE2 and TMPRSS2 and it was largely unaffected by quercetin after 4 h. GAPDH and ACTB were also unchanged in response to quercetin, though expression of both of these genes was >20‐fold and ~300‐fold greater than that of ACE2 and TMPRSS2, respectively. Both were assayed as additional reference genes nonetheless to corroborate the changes in ACE2 and TMPRSS2. The same patterns in gene expression were observed, and there were no significant differences between the quercetin‐induced decreases in ACE2 and TMPRSS2 whether expressed relative to TBP, GAPDH, or ACTB (Table S3).

Since a reduction in both ACE2 and TMPRSS2 mRNA was seen with 10 and 20 μM quercetin in cells cultured for 10 days at an ALI (Figure 1A,B), ACE2 and TMPRSS2 protein expression was measured in cells treated the same way for up to 8 h. Quercetin dose‐dependently decreased ACE2 and TMRPSS2 (*p* = 0.0008 and *p* < 0.0001, respectively). There was a significant decrease in ACE2 with 20 μM quercetin after 4 h (*p* = 0.0092) and 8 h (*p* = 0.0173) (Figure 1E). TMPRSS2 was significantly decreased by quercetin at both 10 μM (*p* = 0.0087) and 20 μM (*p* < 0.0001) after 4 h, but these effects were diminished by 8 h (Figure 1F).

### 3.3 Effects of long‐term quercetin treatment on ACE2 and TMPRSS2 expression in Calu‐3‐ALIs

**FIGURE 2:**
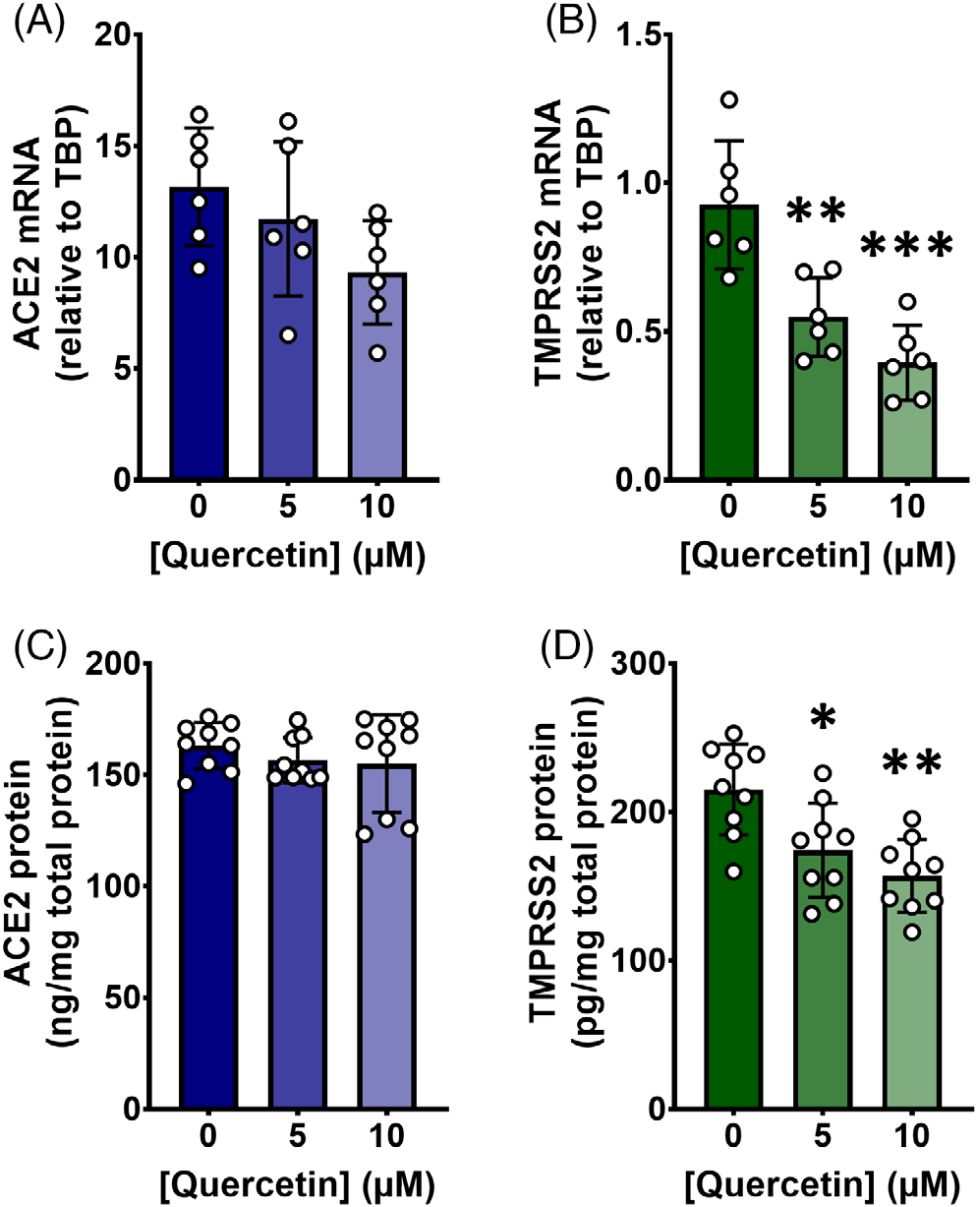
Long‐term quercetin treatment decreases transmembrane protease, serine 2 (TMPRSS2), but not angiotensin‐converting enzyme 2 (ACE2), in Calu‐3‐air–liquid interface (ALI) cells. Calu‐3 cells were cultured on polycarbonate membrane inserts at an ALI for 10 days after reaching confluence. For the final 3 days, cells were treated apically with quercetin or 0.1% (v/v) DMSO vehicle control in serum‐free medium for 1 h twice per day. Cells were harvested for RNA or protein 4 h after the final application of quercetin. For gene expression (A, B), total RNA was isolated and cDNA was synthesized, partitioned, amplified, and analyzed by ddPCR using gene expression assays. Gene expression was quantified as copies per ng mRNA, assuming a 1:1 RT efficiency, and ACE2 (A) and TMPRSS2 (B) target genes are expressed relative to the TBP reference gene. For protein expression (C, D), ACE2 (C) and TMPRSS2 (D) were quantified by ELISA and expressed relative to total protein in the cell lysate. Data were collected independently for each gene/protein from cells treated on duplicate (A, B) or triplicate (C, D) plates across three biological passages. Data are presented as mean ± SD with individual data points representing each cell experiment (*n*/*N* = 6/3 for gene expression, *n*/*N* = 9/3 for protein expression). Significant differences were determined by one‐way ANOVA with post‐hoc Tukey's multiple comparisons. *p < 0.05, **p < 0.01, ***p < 0.001 vs. 0 μM quercetin.

Repeated doses of lower concentrations of quercetin over several days had the same effect as higher doses in the short term on TMPRSS2 mRNA and protein expression. Quercetin dose‐dependently decreased TMPRSS2 mRNA (*p* = 0.006, Figure 2B) and protein (*p* = 0.0046, Figure 2D). However, the longer‐term quercetin treatment had no effect on ACE2 expression at either the transcriptomic (*p* = 0.1097, Figure 2A) or protein (*p* = 0.2723, Figure 2C) levels.

### 3.4 Effects of acute quercetin treatment on expression of ACE2 and TMPRSS2 in PBECs

**FIGURE 3:**
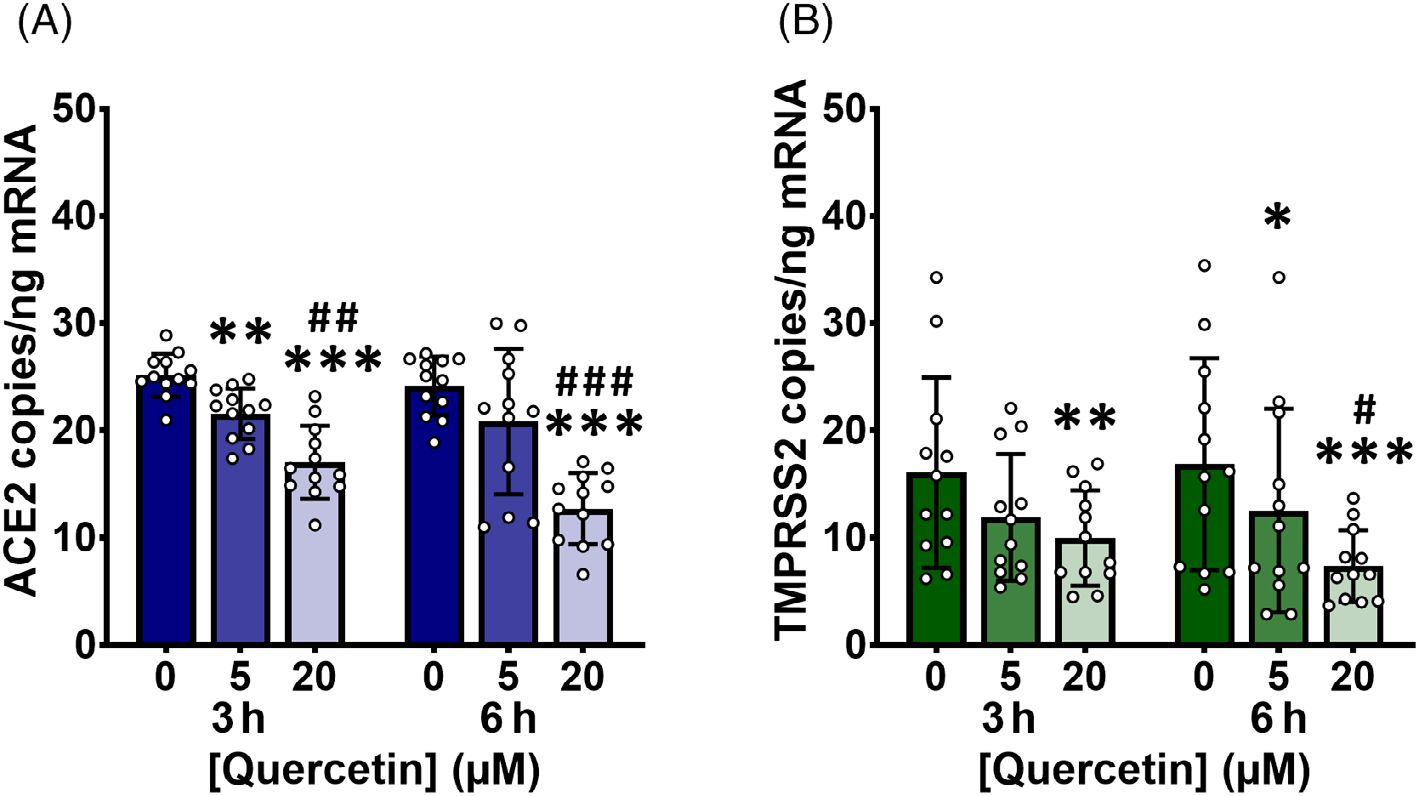
Quercetin decreases angiotensin‐converting enzyme 2 (ACE2) and transmembrane protease, serine 2 (TMPRSS2) in human primary bronchial epithelium cells (PBECs). Human PBECs were isolated and cultured until 80% confluence was reached. Cells were treated with quercetin or 0.1% (v/v) DMSO vehicle control in serum‐free medium for up to 6 h. Total RNA was isolated and cDNA was synthesized, partitioned, amplified, and analyzed by ddPCR using gene expression assays specific to ACE2 (A) or TMPRSS2 (B) and quantified as copies per ng mRNA, assuming a 1:1 RT efficiency. Data were collected independently for each gene from cells originating from four donors, each cultured and treated in triplicate. Data are presented as mean ± SD with individual data points representing each culture (*n*/*N* = 12/4). Significant differences were determined by two‐way ANOVA with post‐hoc Tukey's multiple comparisons. *p < 0.05, **p < 0.01, ***p < 0.001 vs. 0 μM quercetin; ^#^ *p* < 0.05, ^##^ *p* < 0.01, ^###^ *p* < 0.001 vs. 5 μM quercetin.

Mean expression of ACE2 and TMPRSS2 was ~10‐fold lower in primary PBECs than in immortalized Calu‐3 cells cultured at an ALI. Quercetin dose‐dependently decreased ACE2 (*p* < 0.0001) and TMPRSS2 (*p* = 0.0002) at the transcriptomic level in PBECs (Figure 3). There was no main effect of time or treatment × time interaction detected for either gene. However, the decrease in ACE2 with 5 μM quercetin after 3 h was diminished by 6 h (Figure 3A), while the decrease in TMPRSS2 was more significant after 6 h (Figure 3B). Looking at the data from each donor independently, it was evident that quercetin dose‐dependently decreased ACE2 in cells isolated from every donor, while the effect on TMPRSS2 was not observed for each donor, likely due to a larger variation in baseline expression.

### 3.5 Effects of quercetin on SARS‐CoV‐2 infectivity in Calu‐3‐ALIs

**FIGURE 4:**
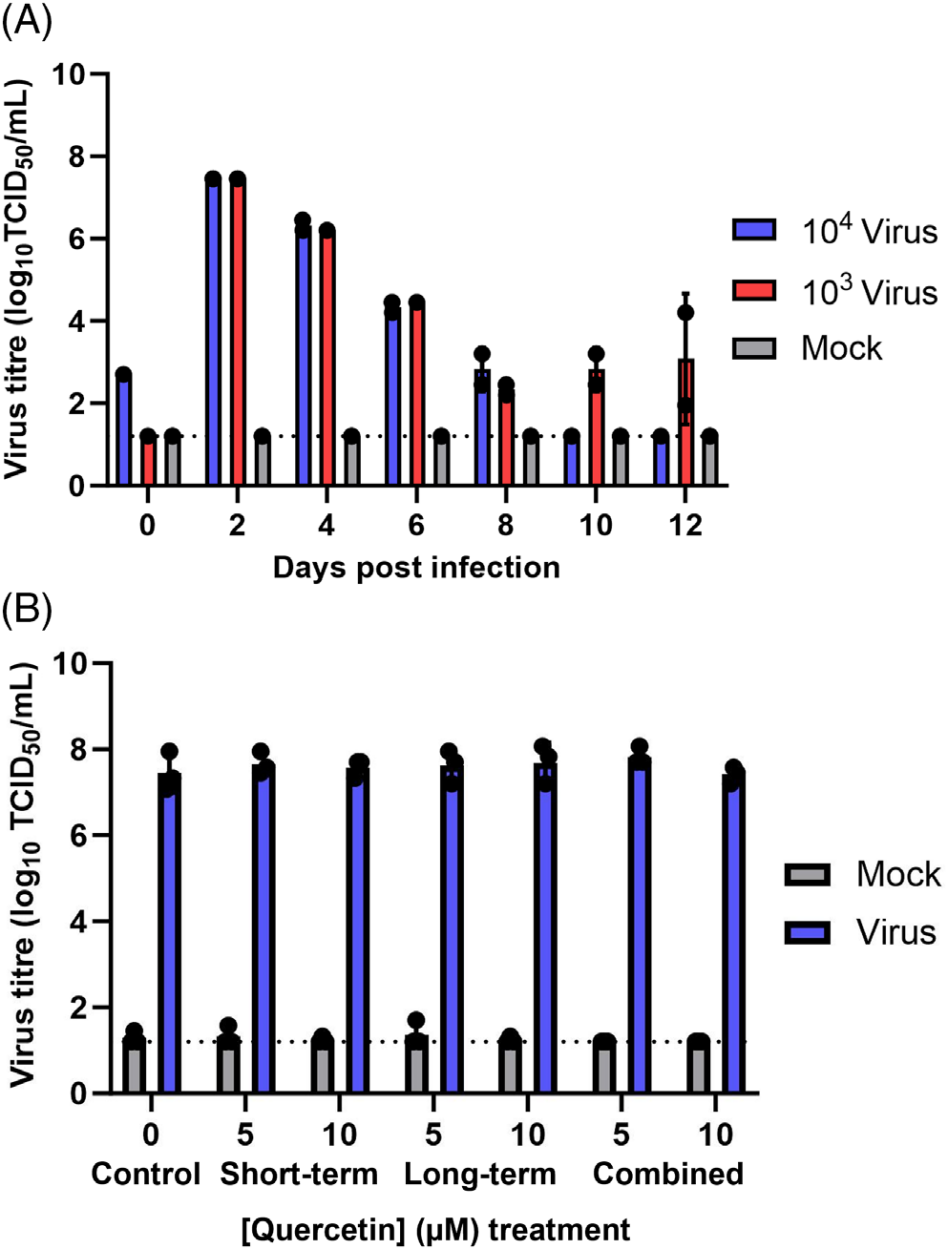
Quercetin did not affect severe acute respiratory syndrome coronavirus 2 (SARS‐CoV‐2) infectivity in Calu‐3‐air–liquid interface (ALI) cells. Calu‐3 cells were cultured on polycarbonate membrane inserts at an ALI for 10 days after reaching confluence. Virus titer was measured for 12 days by infectivity assay in apical washes from Calu‐3‐ALI cells inoculated with SARS‐CoV‐2 at a median tissue culture infectious dose (TCID_50_) of 10^4^ or 10^3^ or with a mock inoculum (A). The effect of quercetin on SARS‐CoV‐2 infectivity in Calu‐3‐ALI cells, directly or indirectly via host entry protein regulation, was assessed (B). All treatments were performed concurrently and cells were inoculated with 10^3^ TCID_50_ SARS‐CoV‐2 or the mock for 1 h, with virus infectivity assayed 2 days post‐infection. For the short‐term treatment, cells were treated apically with quercetin or 0.1% (v/v) DMSO vehicle control in serum‐free medium during the 1 h inoculation only. For the long‐term treatment, cells were pre‐treated apically with quercetin or 0.1% (v/v) DMSO vehicle control in serum‐free medium for 1 h twice per day for the final 3 days of culture and the inoculum applied 3 h after the final quercetin application was removed. For the combined treatment, the long‐term regimen was followed but with quercetin re‐added with the inoculum. The control treatment consisted of only vehicle control applied throughout. Data were collected in quadruplicate from cells across three biological passages (*n*/*N* = 12/3). Bars indicate mean ± standard deviation, dots indicate the mean for each biological passage, and the dotted line indicates the limit of detection.

To determine whether Calu‐3‐ALI cells were permissive to SARS‐CoV‐2 infection, cells were inoculated with 10^3^ or 10^4^ TCID_50_ of the ancestral strain of SARS‐CoV‐2 and infectious virus was measured in apical washes every 2 days up to 12 days post‐infection (dpi). SARS‐CoV‐2 showed robust replication in Vero cells, with peak virus titers of 7.45 log_10_ TCID_50_ achieved at 2 dpi for both 10^3^ and 10^4^ inoculums (Figure 4A). Virus titer steadily decreased at subsequent sampling time‐points, with infectious virus being undetectable in the 10^4^ dose samples at 10 dpi.

To test whether quercetin could modulate SARS‐CoV‐2 infection in Calu‐3‐ALIs, cells were treated with quercetin for 1 h twice per day for 3 days (long‐term treatment), with a single dose of quercetin with the virus (short‐term), or with a combination of the long‐term and short‐term regimens (combined). Calu‐3‐ALIs were inoculated with 10^3^ virus, and infectious virus was assessed at 2 dpi. Vehicle controls reached a mean virus titer of 7.45 log_10_ TCID_50_ at 2 dpi (Figure 4B) but none of the quercetin treatments significantly changed the virus titer at 2 dpi.

## 4 DISCUSSION

In the present study, we aimed to ascertain whether ACE2 and TMPRSS2 were rate‐limiting for SARS‐CoV‐2 viral entry in cultured cells and assess the potential for quercetin to modulate expression of these entry proteins. Based on data from in silico studies that suggested quercetin could interact with ACE2 and/or the ACE2‐spike protein complex, we hypothesized that quercetin could inhibit the protease‐dependent SARS‐CoV‐2 infection route, either directly or via regulation of host entry protein expression.

First, we measured the expression of ACE2 and TMPRSS2 in various human cell lines relevant to SARS‐CoV‐2 infection and COVID‐19. We demonstrated that expression of ACE2 was negligible in human primary endothelial (HUVEC) and immortalized nasal epithelial (RPMI 2650) cells and was very low in human immortalized lung A549 and NCI‐H23 cell lines, although ACE2 increased in mature NCI‐H23 cells. This was despite some previous studies using these cultured cells to investigate effects on ACE2, TMPRSS2, and/or SARS‐CoV‐2 infectivity.^44^, ^45^, ^46^, ^47^ Conversely, HUVEC,^48^ NCI‐H23 and A549 cells,^49^ were transfected to stably express ACE2, given the low basal expression, and it was shown that culturing A549 cells at an ALI for 14 or 21 days induced co‐expression of ACE2 and TMPRSS2 and facilitated in vitro SARS‐CoV‐2 infection.^50^ In the present study, expression of ACE2 in all of these cells was unaffected by exposure to estradiol or apelin‐13, possibly since basal expression was low. TMPRSS2 expression was negligible in HUVEC but present in all airway cell lines, increasing in mature A549 and NCI‐H23 cells, but decreasing with culture time in RPMI 2650 nasal epithelial cells. One study found that ACE2 was increased in A549 cells post‐confluence,^51^ which we validated only for TMPRSS2, and this may explain why others did not detect TMPRSS2 in A549 cells previously.^52^ It has been demonstrated by us and others that SARS‐CoV‐2 infection is dependent on the expression of both ACE2 and TMPRSS2.^2^, ^8^, ^10^, ^11^ SARS‐CoV‐2 can infect other cultured endothelial cells,^25^, ^26^ which would serve as a better model of infectivity than HUVEC, though the latter may still be used for investigating progression of COVID‐19, such as the hyperinflammatory host response.^53^ To date, there has been limited use of immortalized nasal epithelial cell lines for investigation of SARS‐CoV‐2 infectivity in vitro, possibly due to the low levels of ACE2 and TMPRSS2, despite high co‐expression in the nasal epithelium in vivo.^33^ However, we demonstrated previously that primary nasal epithelial cell cultures are a useful tool for such work.^11^, ^54^

ACE2 and TMPRSS2 were highly co‐expressed in Calu‐3 immortalized lung epithelial cells, especially when cultured on a transmembrane support at an ALI for ≥10 days, and this facilitated SARS‐CoV‐2 infection, as we and others have also shown previously.^32^, ^38^, ^42^, ^55^, ^56^ Exposure to estradiol or apelin‐13 upregulated ACE2 and TMPRSS2, as expected.^26^, ^34^, ^35^, ^36^ Acute exposure to quercetin dose‐dependently decreased mRNA expression of ACE2, TMPRSS2, and ADAM17, but not ACE, in Calu‐3 cells cultured at an ALI for 10 days. ACE2 and TMPRSS2 proteins were also transiently decreased. Repeated doses of lower concentrations of quercetin over several days had the same effect as higher doses in the short term on TMPRSS2, but not on ACE2. The acute downregulation of ACE2 and TMPRSS2 mRNA by quercetin was corroborated in human PBECs, despite a ~10‐fold lower basal expression of both genes. It was noted that inter‐individual variation in the expression of TMPRSS2, and the effect on TMPRSS2 by quercetin, among donors was relatively high and this could explain why some individuals are more prone than others to SARS‐CoV‐2 infection and COVID‐19 disease progression. The PBECs were not infected with SARS‐CoV‐2 and a limitation of this study is that the PBECs were not cultured at an ALI, as we have done in other studies investigating SARS‐CoV‐2 infectivity of PBECs and other primary airway epithelial cells.^11^

Similar to the effects of quercetin in Calu‐3‐ALIs and PBECs, a mixture containing quercetin downregulated TMPRSS2 and other androgen‐responsive genes in prostate cancer cells,^57^ and quercetin acts as an antagonist of estrogen receptor‐mediated activities in HeLa cells.^58^ Since estradiol upregulated ACE2 and TMPRSS2 in Calu‐3‐ALIs, the effect of quercetin may be mediated via the estrogen receptor. Conversely, it has been reported that sirtuin 1 (SIRT1) binds to the ACE2 promoter and regulates ACE2 gene expression in response to energy stress via AMP‐activated protein kinase (AMPK), while IL‐1β decreases the binding of SIRT1 to the ACE2 promoter.^59^, ^60^ Quercetin has been suggested in other contexts to increase SIRT1 via AMPK activation,^41^, ^61^ indicating that the molecular mechanism of quercetin action requires further investigation. Here, the quercetin‐induced decrease in ACE2 was transient and expression was unchanged in response to the longer, repeated treatment.

Despite the promising effects on the expression of ACE2 and TMPRSS2, pre‐treatment with quercetin had no effect on SARS‐CoV‐2 infectivity in the Calu‐3‐ALIs. This suggests that ACE2 and TMPRSS2, while essential to the primary protease‐dependent viral infection route, are not rate‐limiting factors in SARS‐CoV‐2 infection in cultured Calu‐3‐ALIs and that basal co‐expression of the entry proteins was so high in this model that even decreasing expression by up to half (Figure 1) was insufficient to prevent viral entry. With less TMPRSS2, viral entry via clathrin‐mediated endocytosis may have been increased.^8^, ^9^ Quercetin also had no effect on viral entry when administered with the SARS‐CoV‐2 inoculum. Despite the in silico evidence,^16^, ^17^, ^24^ this suggests that quercetin does not interact with the ACE2‐viral spike protein complex and/or the physiologically relevant concentrations of quercetin tested were not high enough to see the effect of a direct interaction. Testing quercetin against SARS‐CoV‐2 infectivity at ≤10 μM was in line with the predicted inhibition constant of ~7 μM and recommendations on the bioavailability of quercetin if delivered via a nasal or throat spray, rather than orally.^19^ However, in the study by Kandeil et al.,^62^ 18.2 μM quercetin reduced SARS‐CoV‐2 plaques in Vero E6 cells by half via viricidal effects and inhibition of viral replication. Further, our data are contrary to the antiviral properties of quercetin against other viruses, such as influenza A (H1N1), hepatitis B and C, and human immunodeficiency virus 1.^63^

While quercetin may not affect SARS‐CoV‐2 infectivity in Calu‐3‐ALIs, there is evidence that this flavonoid can regulate COVID‐19 progression. Notably, a recent randomized clinical trial demonstrated a therapeutic effect of quercetin in outpatients with early‐stage mild‐to‐moderate symptoms of COVID‐19. Patients received either standard care plus an oral quercetin supplement (500 mg) or standard care alone (*n* = 50 per group). After 1 week of quercetin treatment, 68% of patients tested negative for SARS‐CoV‐2 and 52% of patients showed no further COVID‐19 symptoms, compared to only 24% of patients testing negative or without symptoms in the standard care group.^30^ These data suggest that quercetin increased clearance of SARS‐CoV‐2 and modulated the host inflammatory response. The longer‐term quercetin treatment in the current study, whereby TMPRSS2 was downregulated after 3 days but ACE2 was only transiently downregulated, is consistent with the findings in humans. A decreased level of TMPRSS2 may reduce the rate of cell‐to‐cell SARS‐CoV‐2 transmission and immunopathology,^64^ thereby decreasing COVID‐19 progression and inter‐individual spread. Concurrently, ACE2 is involved in the regulation of the host inflammatory response. It seems that SARS‐CoV‐2 entry into cells downregulates ACE2^65^ and this contributes to COVID‐19 pathophysiology via instigation of the hyperinflammatory cytokine storm and manifestation of cardiovascular disruption,^66^ while maintenance of ACE2 levels could help to alleviate this. Further, quercetin has been shown to modulate a broad range of immune mechanisms,^67^ which likely facilitated viral clearance and improved outcomes in humans.^30^

While regulation of ACE2 and TMPRSS2 has the potential to modulate COVID‐19, there are some potential implications to such gene regulation by exogenous compounds, due to the various physiological roles of these enzymes. A reduction in ACE2 may lead to an increase in ACE activity, which could result in elevated blood pressure and an increased risk of cardiovascular disease, while reduced ACE2 in lung has been implicated in the pathogenesis of pulmonary arterial hypertension, via an imbalance in the renin–angiotensin–aldosterone system system.^68^, ^69^ In the present study, ACE2 was maintained during the longer‐term exposure to quercetin and, with no effects on the expression of ACE, this may partially explain the faster resolution of symptoms in humans with mild COVID‐19 when taking quercetin supplements.^30^ Furthermore, we demonstrate that quercetin acutely downregulates ADAM17. While ADAM17 does not facilitate SARS‐CoV‐2 entry,^3^, ^4^ the protease may mediate COVID‐19 severity via pathological activation through intracellular SARS‐CoV‐2 accumulation, leading to enhanced ADAM17 activity. This in turn increases shedding of ACE2 and release of inflammatory cytokines (the cytokine storm), while activation of ADAM17 promotes SARS‐CoV‐2 replication.^5^ A reduced expression of ADAM17 in response to quercetin could reduce SARS‐Cov‐2 replication, increase clearance, and alleviate symptoms, and this warrants further investigation. Beyond COVID‐19, reduced ADAM17 expression in lung may have promising implications in *KRAS* mutation‐driven lung adenocarcinoma.^70^

## 5 CONCLUSIONS

Quercetin acutely downregulates ACE2 and TMPRSS2 mRNA and protein in Calu‐3 cells cultured at an ALI, and TMPRSS2 remains downregulated in response to longer‐term repeated dosing, while ACE2 recovers. Acute exposure to quercetin also decreased ADAM17 mRNA, but not ACE, and this requires further investigation. The downregulation of ACE2 and/or TMPRSS2 was insufficient to decrease SARS‐CoV‐2 infectivity, nor did quercetin act as a direct inhibitor of viral entry, demonstrating that a significant decrease in the expression of ACE2 and TMPRSS2 by a promising prophylactic candidate may not translate to infection prevention. However, the downregulation of TMPRSS2, and not ACE2, in response to a chronic exposure of quercetin is promising for COVID‐19 outcomes. This effect may reduce viral load and transmission, slow disease progression, and limit complications and severity, without impacting the regulatory function of ACE2 on the renin–angiotensin–aldosterone system nor the hyperinflammatory response associated with ACE2 dysregulation.

## AUTHOR CONTRIBUTIONS

Study conception and design, data collection, analysis and interpretation of results, writing the first draft of the manuscript, and manuscript review: MJH. Study conception and design, data collection, and manuscript review: EB. Study design, data collection, analysis and interpretation of results, and manuscript preparation and review: MJG. Human primary bronchial epithelial cell isolation and culture, and manuscript review: BJT. Study design and manuscript review: KS. Study conception and design, interpretation of results, manuscript review and funding: GW.

## Supporting information


**Table S1.** Cell viability, assessed by Trypan Blue exclusion, and transepithelial electrical resistance (TEER) values of Calu‐3‐ALIs, differentiated for up to 20 days post‐confluence. Quercetin treatment did not affect viability or cell epithelium integrity, as determined by TEER values.
**Table S2.** Quercetin acutely decreases ACE2, TMPRSS2 and ADAM17 mRNA in Calu‐3‐ALI cells differentiated for 10 days, and ACE2 and TMPRSS2 mRNA in Calu‐3‐ALI cells differentiated for 20 days. Absolute copy numbers and gene expression relative to TBP are shown. Absolute expression of ACE2, TMPRSS2 and ADAM17 was also dose‐dependently decreased, while TBP was unchanged. Gene expression assays for ACE2 and TMPRSS2 were first conducted on Calu‐3 cells cultured at an ALI for 20 days, since ACE2 expression was at its highest in this model and quercetin dose‐dependently decreased expression of both, corroborating the results seen in cells cultured for 10 days.
**Table S3.** ACTB and GAPDH were used as reference genes, in addition to TBP, in Calu‐3‐ALI cells differentiated for 10 and 20 days. TBP was selected as the reference gene because, of those screened, its expression was closest in range to that of both ACE2 and TMPRSS2 and it was unaffected by quercetin after 4 h. GAPDH and ACTB were also largely unchanged in response to quercetin and corroborate the changes in ACE2 and TMPRSS2.

## Data Availability

The data that support the findings of this study are available from the corresponding author upon reasonable request.
